# Temporal Variability and Influence of Measurement Conditions of AI‐Based Atrial Fibrillation Risk Estimation

**DOI:** 10.1002/joa3.70280

**Published:** 2026-02-05

**Authors:** Satomi Hamada, Miki Amemiya, Mie Ochida, Susumu Tao, Iwanari Kawamura, Tetsuo Sasano

**Affiliations:** ^1^ Department of Cardiovascular Medicine Institute of Science Tokyo Tokyo Japan; ^2^ Department of Clinical Laboratory Institute of Science Tokyo Hospital Tokyo Japan

**Keywords:** artificial intelligence, atrial fibrillation, electrocardiography, reproducibility, risk stratification

## Abstract

**Background:**

Although artificial intelligence (AI) has been developed to identify patients with paroxysmal atrial fibrillation (PAF) during sinus rhythm, information on its variability remains limited. We evaluated the reproducibility and effect of recording condition on the estimation of AF risk using an electrocardiography (ECG) machine equipped with an AI‐based program.

**Methods:**

We extracted two ECG data from a single ECG test in 149 patients to evaluate reproducibility within 4 min. We also recorded ECG signals under 12 conditions (standard, two conditions shifting precordial electrodes, five conditions moving limb electrodes to the torso, three conditions contaminating noise, and reproducibility over 15 min) in 30 participants to evaluate changes from the standard. The results of the AF risk estimation are expressed at four levels.

**Results:**

The rate of participants within one level of error was 95% for reproducibility within 4 min and 87% for reproducibility over 15 min. Shifting the precordial electrodes upward or downward and replacing the left leg electrode with the torso electrode frequently caused a two‐ or three‐level change. In clinical information, increased brain natriuretic peptide tended to increase the variability.

**Conclusions:**

The AF risk estimated by the AI‐based program exhibited temporal variability. Shifting the precordial electrodes influenced AI‐based AF risk estimation.

## Graphical abstract

The atrial fibrillation (AF) risk estimated by artificial intelligence‐based program installed in electrocardiography (ECG) machine exhibited temporal variability. Reproducibility within one level of error was 100% for the same ECG, 95% for reproducibility within 4 min interval, and 87% for reproducibility over 15 min interval.

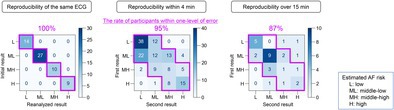

## 1 Introduction

Atrial fibrillation (AF) is often detected as paroxysmal atrial fibrillation (PAF), which can develop into persistent, long‐standing persistent, and permanent AF [1, 2, 3]. Although the early detection of PAF is important, conventional 12‐lead electrocardiogram (ECG) for 10 s has been shown to identify fewer PAF cases than long‐term ECG monitoring [4]. Recently, artificial intelligence (AI), which identifies patients with PAF during sinus rhythm using an ECG of 10 s duration, has been developed [5, 6]. We recently developed an edge AI model [6], and an ECG machine equipped with the edge AI model is commercially available.

One issue in the application of AI is the reduction in performance caused by a domain shift. The causes of the domain shift are believed to include differences between the populations of training data and evaluation data, including the instability of data acquisition. In the case of ECG, the use of different ECG machines, placement of electrodes, and changes in the environment during recording can contribute to domain shifts. On the other hand, ECG data are known to have physiological and pathophysiological variabilities, and evaluations of these variabilities have been conducted. In AI analysis, it is conceivable that variability has an impact; however, investigation into this aspect remains minimal.

Although several studies using 12‐lead ECG have been published, information on their reproducibility and stability remains limited. ECG is recognized as a reproducible technique, and it is known that the change in electrode placement varies the waveform, and that inaccurate electrode placement can result in misdiagnosis [7]. Noises also alter ECG waveforms. AI algorithms for the prediction of PAF often use raw ECG signals [8, 9], thus they may be affected by changes in the ECG that cannot be detected by visual inspection.

In this study, we evaluated the reproducibility and the effects of electrode placement and noise contamination on the estimation of AF risk using a commercially available ECG machine equipped with an AI‐based program.

## 2 Methods

### 2.1 Study Design and Participants

We enrolled 180 participants, including 170 patients with cardiovascular disease at the Institute of Science Tokyo Hospital and 10 healthy volunteers. Inclusion criteria were an age of 18 years or older and being in sinus rhythm.

This study evaluated two potential factors influencing AI‐based assessment of AF risk. The first was the intrinsic temporal variability, and the second was the electrode position and noise contamination. We performed ECG recordings under numerous conditions for the first and second purposes (*Study 1*) and extracted multiple ECGs recorded from the same participant over a very short period of time for the first purpose (*Study 2*). We then evaluated the ECG using AI‐based AF risk estimation.

Study 1 included 31 participants: 21 patients with cardiovascular disease and 10 healthy volunteers. Patients with a history of open‐chest surgery or cardiac implantable device implantation were excluded from the study. Study 2 included 149 patients who underwent two ECG recordings within 4 min in the clinical laboratory at the Institute of Science Tokyo Hospital from April 2024 to December 2024. The cases who had ECG without sinus rhythm were excluded. This study was approved by the Ethics Committee of the Institute of Science, Tokyo, Japan (No. I2024‐046).

### 2.2 ECG Recording Protocol in Study 1

In Study 1, ECG recording was conducted using CardiMax9 FCP‐9900Ai (Fukuda Denshi Co. LTD, Tokyo, Japan) with a sampling rate of 500 Hz, a resolution of 4.88 μV/LSB (least significant bit), and a band‐pass filter of 0.05–250 Hz, according to the manufacturer's instruction. ECGs were recorded during sinus rhythm for 10 s. Disposable electrodes with adhesive gels (NIPRODE III TEE‐173DN; Fukuda Denshi) were used.

We recorded 12‐lead ECGs under 12 conditions (Table 1) and performed AI‐based AF risk estimation. The ECGs under the 12 conditions were recorded at minimum intervals. Each ECG was recorded during sinus rhythm for 10 s. If the recorded ECG demonstrated transient arrhythmia that disturbed the AF risk estimation (e.g., premature contraction), we recorded another ECG. If arrhythmia occurred frequently and the measurement required more than 20 min, we excluded the participants from this study.

**TABLE 1:** Conditions for ECG recording in Study 1.

No.	Abbreviation	Electrode placement	Noise contamination
1	Standard‐1	Standard 12‐lead ECG	At rest
2	Precordial‐up	Precordial electrodes were shifted upward one intercostal space	At rest
3	Precordial‐down	Precordial electrodes were shifted downward one intercostal space	At rest
4	RA‐torso	RA electrode was placed on the right infraclavicular fossa	At rest
5	LA‐torso	LA electrode was placed on the left infraclavicular fossa	At rest
6	RL‐torso	RL electrode was placed on the right iliac fossa	At rest
7	LL‐torso	LL electrode was placed on the left iliac fossa	At rest
8	All‐torso	Combination of No. 4–7	At rest
9	Drift‐noise	Standard 12‐lead ECG	Deep breathing
10	EMG‐noise	Standard 12‐lead ECG	Hand gripping
11	AC‐noise	Standard 12‐lead ECG	Applying external AC noise
12	Standard‐2	Standard 12‐lead ECG	At rest

Abbreviations: AC, alternating current; EMG, electromyogram.

**FIGURE 1:**
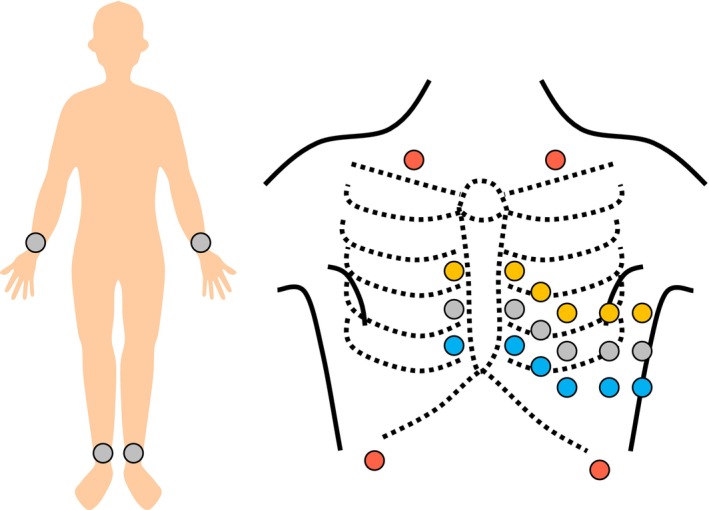
Electrode placement. A total of 26 disposable electrodes were attached to the participants and connected to ECG cables in sequence. The electrodes were placed in a standard 12‐lead ECG position (gray), one intercostal space above the precordial electrodes (yellow), one intercostal space below the precordial electrodes (blue), and Mason‐Liker lead position (red).

Initially, a standard 12‐lead ECG (No. 1) was performed at rest. This was used as a reference. We then shifted the precordial electrodes upward or downward by one intercostal space (No. 2–3) and moved the limb electrodes to where the limbs joined, such as the Mason‐Likar lead position (No. 4–8). All electrodes were attached at the beginning of the ECG measurement, and the connections of the ECG cables were changed (Figure 1). In the study of noise contamination, we recorded ECG, including drift noise by deep breathing, electromyogram (EMG) noise by both hand gripping, and alternating current (AC) noise by placing electrical devices closer to the ankles of the participants (No. 9–11). We selected these conditions because misplacement of precordial electrodes can occur frequently (No. 2–3) [10, 11], and the limb electrodes have to be attached to the torso in clinical situations where they cannot be attached to the limbs (No. 4–8). In addition, we examined typical low‐ and high‐frequency noises (No. 9–11) [12]. Finally, we recorded a standard 12‐lead ECG at rest (No. 12) to examine the reproducibility over 15 min. In addition, we reanalyzed the stored ECG data and compared the reanalyzed results of the AI‐based AF risk estimation with the initial results.

### 2.3 ECG Extraction in Study 2

ECG data recorded in the clinical laboratory of the Institute of Science Tokyo Hospital were extracted for Study 2. ECG recordings in the clinical laboratory were performed using a CardiMax9 FCP‐9900 (Fukuda Denshi). We obtained two ECGs from the same patient within 4 min. The extracted ECG data were transferred to a CardiMax9 FCP‐9900Ai for AI‐based AF risk estimation. Reproducibility was evaluated by comparing the AI risk between the two ECGs.

### 2.4 AI‐Based Atrial Fibrillation Risk Estimation

The program built into the CardiMax9 FCP‐9900Ai estimated the AF risk, which indicates the probability that a participant has already developed PAF. The results are expressed at four levels: low (L), middle‐low (ML), middle‐high (MH), and high (H).

According to the manufacturer's instruction, the participants younger than 40 years old, the participants with specific disease or treatment (mitral stenosis, history of pacemaker implantation, history of catheter ablation, history of heart surgery, history of artificial valve replacement, or use of anti‐arrhythmic drugs), and the abnormal ECGs defined by following Minnesota Code (6‐1, 6‐2‐1, 6‐2‐2, 6‐2‐3, 6‐8, 8‐1‐1, 8‐1‐2, 8‐1‐3, 8‐1‐4, 8‐1‐5, 8‐1‐6, 8‐1‐7, 8‐1‐8, 8‐2‐1, 8‐2‐2, 8‐2‐3, 8‐3‐1, 8‐3‐2, 8‐4‐1, 8‐4‐2, 8‐4‐3, 8‐5, 8‐6‐1, 8‐6‐2, 8‐6‐3, 8‐6‐4, 8‐7‐1, 8‐7‐2, 8‐8‐1, 8‐8‐2, 8‐9‐2, 8‐9‐4, 8‐9‐9, 9‐6‐2, 9‐8‐3, 9‐8‐4, 9‐8‐8, for example, premature contractions, sustained and non‐sustained tachycardias, bradycardia, atrioventricular block, ectopic atrial rhythm, atrial fibrillation and flutter, artificial pacemaker rhythm, unsatisfactory record) were not eligible for AI‐based AF risk estimation. In this study, we excluded the abnormal ECG traces defined above. When the participant was younger than 40 years, we proceeded with the AI‐based estimation by inputting the participant's age as 40 years. Patients with disease or treatment that were not eligible for AI‐based AF risk estimation were included in the analysis.

### 2.5 Background Information

Information regarding age, sex, present illness, and medical history was obtained from patients through medical records, and from volunteers through interviews. Height and weight were obtained in all participants in Study 1. ECG measurements were obtained from the first result (Standard‐1 in Study 1 and ECG‐1 in Study 2) for each participant. Heart rate, PR interval, QRS duration, QT interval, QTc interval by Bazzet's formula, QTc interval by Fridericia's formula, QRS axis, the higher R amplitude in either lead V_5_ or V_6_, S amplitude in lead V_1_, the higher R amplitude in either lead V_5_ or V_6_ + S amplitude in lead V_1_ were measured by automatic analysis. P wave amplitude in lead II, P wave duration in lead II, and Morris index were measured by visual inspection. Whether the P wave was biphasic was also determined visually. Echocardiography and Holter ECG within 3 years from ECG measurement and brain natriuretic peptide (BNP) level measured on the same day as ECG measurement were used for analysis.

### 2.6 Statistical Analysis

Statistical analyses were conducted using the Python library statistical packages (pandas 1.5.3 and scikit‐learn 1.3.0). The agreement between the two results of AF risk estimation was evaluated using quadratic‐weighted kappa with a 95% confidence interval, which was estimated using a bootstrap sampling (10 000 iterations). Continuous variables were expressed as mean ± standard deviation, median [range], or value [95% confidence interval] as appropriate.

## 3 Results

### 3.1 Temporal Variability of AI‐Based AF Risk Estimation

**FIGURE 2:**
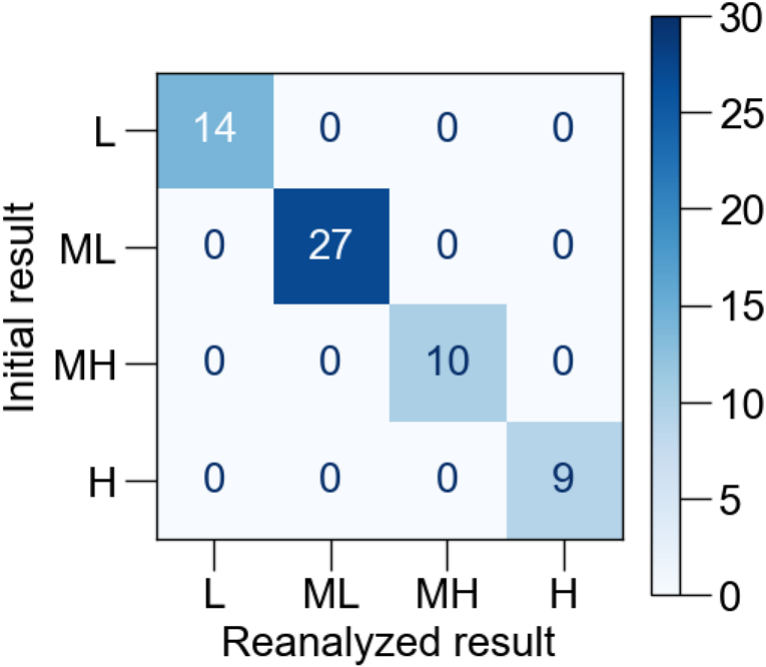
Confusion matrix for the evaluation of reproducibility of AI model. To confirm the instability of the algorithm, stored ECG data were reanalyzed and compared with initial results. The results of the AF risk estimation were expressed as four levels: Low (L), middle‐low (ML), middle‐high (MH), and high (H).

Of the 31 participants enrolled in Study 1, one was excluded because of frequent premature contractions. Therefore, we performed AF risk estimation in 30 participants. The background characteristics of the participants are presented in Table 2. At first, we confirmed the reproducibility of the AI model using the same ECG data from a total of 60 ECG recordings (Standard‐1 and Standard‐2 in Study 1). We converted the results of AF risk estimation into the following numerical order: 0 for L, 1 for ML, 2 for MH, and 3 for H. All of the reanalyzed results showed the same results as the initial ones (Figure 2).

**TABLE 2:** Background characteristics of the participants in Study 1.

Age (years old)	55 ± 18
Male	11 (37)
Height (cm)	162.5 ± 9.6
Weight (kg)	58.9 ± 11.8
Body mass index	22.2 ± 3.8
Valvular heart disease	3 (10)
Hypertension	9 (30)
Diabetes mellitus	1 (3)
Chronic kidney disease	3 (10)
Paroxysmal atrial fibrillation	2 (7)
Other arrhythmia	6 (20)
Ischemic heart disease	2 (7)
Left ventricular hypokinesis	3 (10)
Cerebral infarction	1 (3)

*Note:* Data are presented as mean ± standard deviation or *n* (%).

**FIGURE 3:**
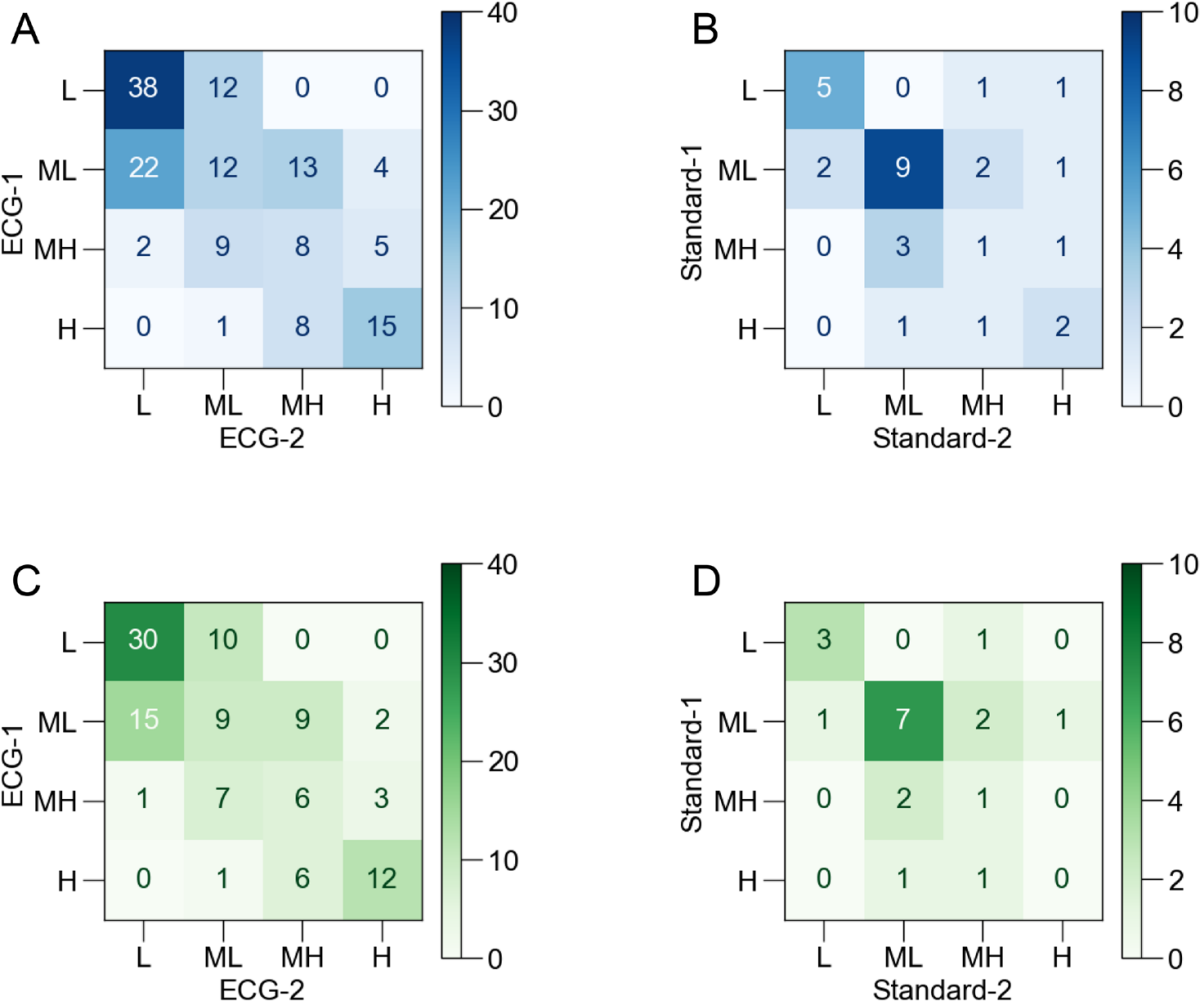
Confusion matrices for the evaluation of reproducibility. The AF risk was estimated using two ECG recordings in all participants (A, B) and only in participants who were eligible for AI‐based AF risk estimation (C, D). ECG recordings were taken within 4 min (A, C) and approximately 15 min (B, D). The results of the AF risk estimation were expressed as four levels: Low (L), middle‐low (ML), middle‐high (MH), and high (H).

**FIGURE 4:**
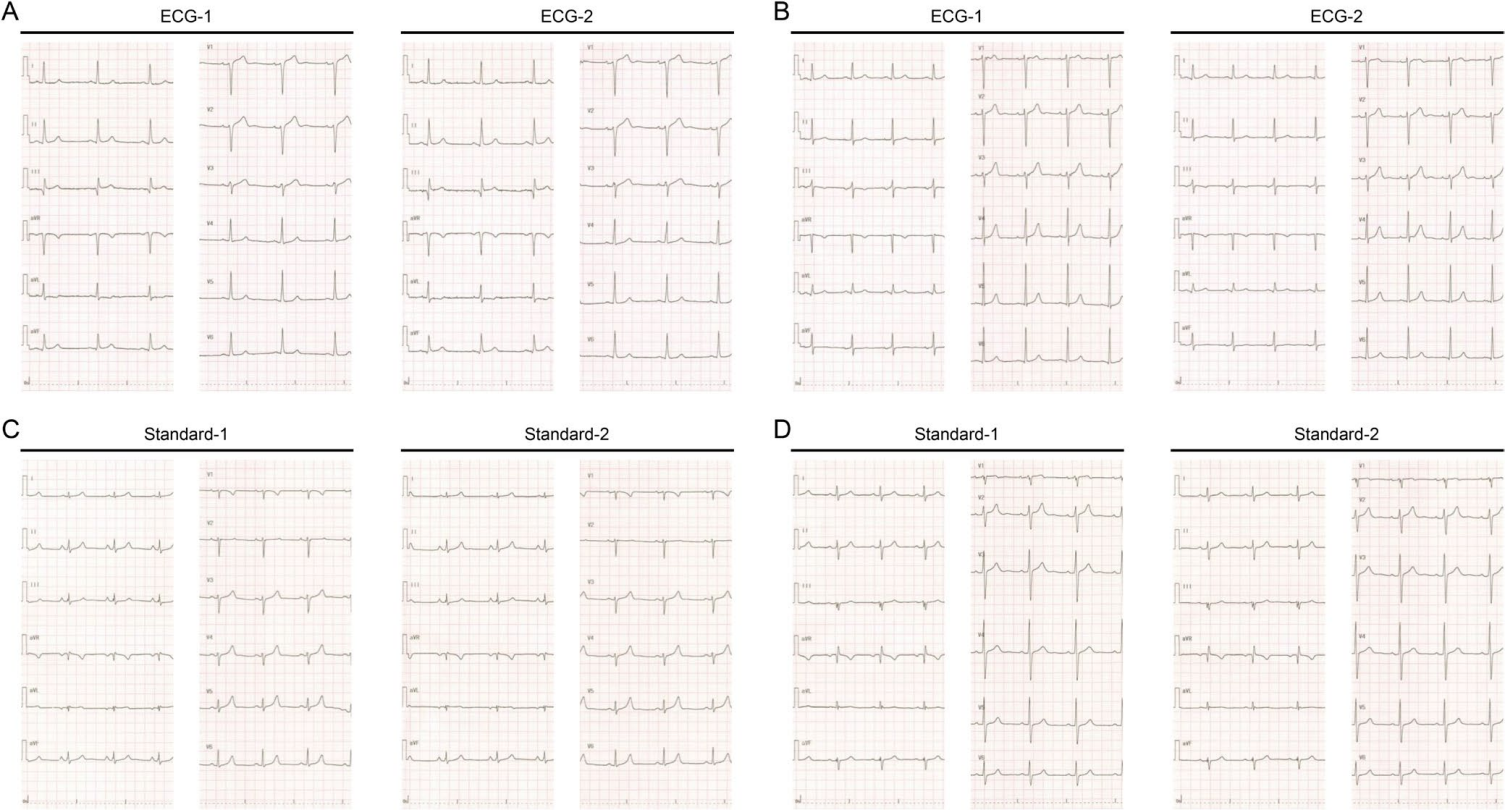
Representative ECG pairs with two‐ or three‐level change in estimated AF risk. Four pairs of ECGs from four participants who showed two‐ or three‐level changes for the reproducibility within 4 min (A, B) and for the reproducibility over 15 min (C, D). Left panels show the first ECG, and right panels show the second ECG. (A) An 81‐year‐old male patient with old myocardial infarction, heart failure, hypertension, diabetes mellitus, chronic kidney disease, and right pelvic lymph node infection. AF risk was ML for ECG‐1 and H for ECG‐2. (B) A 63‐year‐old male patient with prostate cancer and intraocular malignant lymphoma. AF risk was MH for ECG‐1 and L for ECG‐2. (C) A 28‐year‐old female volunteer using a low‐dose contraceptive pill. AF risk was L for Standard‐1 and H for Standard‐2. (D) A 47‐year‐old male patient with premature ventricular complex and left ventricular hypokinesis. AF risk was L for Standard‐1 and MH for Standard‐2. The results of the AF risk estimation were expressed as four levels: Low (L), middle‐low (ML), middle‐high (MH), and high (H).

In next, two ECG traces (ECG‐1 and ECG‐2) recorded in single ECG tests were compared to evaluate very short‐term reproducibility in 149 patients (64 ± 16 years, 86 males) in Study 2. A comparison of AF risk estimations between ECG‐1 and ECG‐2 is illustrated in Figure 3A. The estimated AF risks demonstrated no change in 73 participants (49%), one‐level change in 69 participants (46%), two‐level change in 7 participants (5%), and three‐level change in 0 participants (0%). In total, 95% of the participants had errors within one level of error. The quadratic‐weighted kappa value was 0.72 [0.64–0.79] in all participants. Representative ECG pairs which exhibited two‐level changes are shown in Figure 4A,B.

We also compared Standard‐1 and Standard‐2 in Study 1 to evaluate short‐term reproducibility. The time difference between the recorded time of Standard‐1 and those of Standard‐2 ranged from 12 to 19 min (mean: 15 min). The confusion matrices of the estimated AF risk levels are shown in Figure 3B. AF risk demonstrated no change in 17 participants (57%), one‐level change in 9 participants (30%), two‐level change in 3 participants (10%), and three‐level change in 1 participant (3%). In total, 87% of the participants had errors within one‐level of error. The quadratic‐weighted kappa value was 0.47 [0.07–0.77] in all participants. Representative ECG pairs which exhibited two‐ or three‐level changes are shown in Figure 4C,D.

In order to exclude the influence of diseases and treatments which were not eligible for AI‐based AF risk estimation, we also evaluated the reproducibility only in participants who were eligible for AI‐based AF risk estimation. Of 111 eligible participants in Study 2, AF risk demonstrated no change in 57 participants (51%), one‐level change in 50 participants (45%), two‐level change in 4 participants (4%), and three‐level change in 0 participants (0%). As a result, 96% of the participants had errors within one‐level of error (Figure 3C), and the quadratic‐weighted kappa value was 0.75 [0.66–0.82]. Of 20 eligible participants in Study 1, AF risk demonstrated no change in 11 participants (55%), one‐level change in 6 participants (30%), two‐level change in 3 participants (15%), and three‐level change in 0 participants (0%). Eighty‐five % of the participants had errors within one‐level of error (Figure 3D), and the quadratic‐weighted kappa value was 0.34 [−0.09–0.68]. These results indicated that the eligibility for AI‐based AF risk estimation did not influence the reproducibility. We thus included all participants in following analyses.

We evaluated the patient characteristics to examine the factors affecting reproducibility. Four participants who had two‐ or three‐level changes in Study 1 had the following characteristics: a 28‐year‐old female volunteer using low‐dose contraceptive pill; a 41‐year‐old male patient with left ventricular hypokinesis, hypertension, Crohn's disease, portal hypertension, splenomegaly, esophageal varices, and suspected primary sclerosing cholangitis; a 47‐year‐old male patient with premature ventricular complex and left ventricular hypokinesis; and a 54‐year‐old male patient with hypertension, diabetes mellitus, dyslipidemia, dizziness, and history of cerebral infarction. Of note, one case was not eligible for the analysis by AI model according to the manufacturer's instruction (< 40 years old), and 2 cases had left ventricular hypokinesis. Regarding seven participants who had a two‐level change in Study 2, we could not find a specific underlying disease.

We then explored electrocardiographic features related to the level change in AF risk assessment. Measurements of ECG waveforms were summarized against the magnitude of level change in estimated AF risk in very short‐term reproducibility (Study 2) in Table 3, and those in short‐term reproducibility (Study 1) in Table 4. No significant differences in ECG measurements were observed among the three groups.

**TABLE 3:** Background characteristics against the magnitude of level change in estimated AF risk in very short‐term reproducibility.

	Study 2 (*N* = 149)		
	Zero‐level difference	One‐level difference	Two‐level difference
Age (years)	68 [19–88]	66 [22–87]	74 [29–84]
Male, *N* (%)	40 (54)	40 (58)	6 (86)
Not eligible for AI‐based AF risk estimation, due to age < 40 years, *N* (%)	9 (12)	3 (4)	1 (14)
Not eligible for AI‐based AF risk estimation, due to disease or treatment, *N* (%)	9 (12)	16 (23)	2 (29)

ECG	(*N* = 73)	(*N* = 69)	(*N* = 7)
Heart rate (bpm)	70 [48–98]	72 [51–126]	74 [56–90]
PR interval (s)	0.17 [0.09–0.26]	0.17 [0.12–0.28]	0.15 [0.15–0.18]
QRS duration (s)	0.10 [0.08–0.17]	0.10 [0.09–0.20]	0.10 [0.09–0.11]
QT interval (s)	0.39 [0.34–0.51]	0.39 [0.31–0.51]	0.38 [0.35–0.43]
QTc interval (Bazzet) (s)	0.43 [0.37–0.50]	0.43 [0.38–0.56]	0.42 [0.38–0.44]
QTc interval (Fridericia) (s)	0.42 [0.37–0.50]	0.42 [0.37–0.55]	0.41 [0.38–0.42]
QRS axis (°)	46 [−86–92]	43 [−140–175]	22 [−29–70]
R amplitude in lead V_5_ or V_6_ (mV)	1.44 [0.69–2.76]	1.33 [0.35–2.99]	1.49 [0.73–2.10]
S amplitude in lead V_1_ (mV)	0.83 [0.00–2.50]	0.81 [0.00–3.61]	0.73 [0.12–1.63]
R amplitude in lead V_5_ or V_6_ + S amplitude in lead V_1_ (mV)	2.30 [0.76–4.93]	2.21 [0.39–5.17]	2.22 [0.97–3.62]
P wave amplitude in lead II (mV)	0.10 [0.00–0.20]	0.10 [0.05–0.25]	0.10 [0.05–0.20]
P wave duration in lead II (s)	0.10 [0.04–0.12]	0.10 [0.06–0.12]	0.08 [0.04–0.10]
Morris index (s·mm)	0.02 [0.00–0.12]	0.02 [0.00–0.08]	0.02 [0.00–0.06]
Notched P wave in lead II, *N* (%)	1 (1)	3 (4)	1 (14)

Blood test	(*N* = 14)	(*N* = 12)	(*N* = 2)
BNP (pg/mL)	44.9 [7.2–152.3]	65.2 [9.8–429.7]	113.2 [47.9–178.4]

Echocardiography	(*N* = 20)	(*N* = 26)	(*N* = 4)
Normal left ventricular contraction, *N* (%)	14 (74)	17 (65)	3 (75)
Ejection fraction (%)	66 [38–74]	65 [34–76]	63 [50–75]
Left atrial dimension (mm)	37.7 [27.0–54.2]	38.8 [25.6–54.6]	38.7 [31.4–46.0]
E/A ratio	0.87 [0.39–2.27]	0.82 [0.48–1.68]	0.74 [0.62–1.08]
Deceleration time of E velocity (ms)	210.0 [125.0–324.2]	241.3 [1.0–433.3]	244.3 [169.6–317.0]
E velocity (m/s)	0.72 [0.34–1.01]	0.65 [0.36–1.52]	0.57 [0.37–0.77]
A velocity (m/s)	0.74 [0.32–1.25]	0.70 [0.38–1.77]	0.65 [0.49–1.25]
E/e’ ratio (septal)	11 [5–16]	12 [7–38]	15 [13–16]
e’ velocity (septal) (m/s)	0.06 [0.03–0.13]	0.05 [0.03–0.09]	0.04 [0.04–0.05]

Holter ECG	(*N* = 8)	(*N* = 6)	(*N* = 1)
Number of normal beats/total heartbeats (%)	1.00 [0.88–1.00]	1.00 [0.95–1.00]	1.00 [1.00–1.00]
Number of supraventricular ectopies/total heartbeats (%)	0.00 [0.00–0.01]	0.00 [0.00–0.00]	0.00 [0.00–0.00]
Number of ventricular ectopies/total heartbeats (%)	0.00 [0.00–0.12]	0.00 [0.00–0.05]	0.00 [0.00–0.00]
AF burden/recording time (%)	0.00 [0.00–0.41]	0.00 [0.00–0.00]	0.71 [0.71–0.71]

*Note:* Data are presented as median [range] or *N* (%).

**TABLE 4:** Background characteristics against the magnitude of level change in estimated AF risk in short‐term reproducibility.

	Study 1 (*N* = 30)		
	Zero‐level difference	One‐level difference	Two or three‐level difference
Age (years)	55 [25–87]	62 [32–76]	44 [28–54]
Male, *N* (%)	5 (29)	3 (33)	3 (75)
Not eligible for AI‐based AF risk estimation, due to age < 40 years, *N* (%)	4 (24)	1 (11)	1 (25)
Not eligible for AI‐based AF risk estimation, due to disease or treatment, *N* (%)	2 (12)	2 (22)	0 (0)

ECG	(*N* = 17)	(*N* = 9)	(*N* = 4)
Heart rate (bpm)	61 [49–78]	62 [55–67]	66 [57–85]
PR interval (s)	0.16 [0.12–0.22]	0.16 [0.13–0.23]	0.15 [0.14–0.17]
QRS duration (s)	0.09 [0.08–0.18]	0.09 [0.08–0.12]	0.10 [0.10–0.11]
QT interval (s)	0.43 [0.37–0.45]	0.41 [0.38–0.44]	0.41 [0.39–0.41]
QTc interval (Bazzet) (s)	0.42 [0.40–0.46]	0.42 [0.39–0.45]	0.43 [0.40–0.46]
QTc interval (Fridericia) (s)	0.42 [0.40–0.45]	0.41 [0.39–0.45]	0.42 [0.40–0.44]
QRS axis (°)	54 [−32–109]	59 [−4–85]	41 [−58–64]
R amplitude in lead V_5_ or V_6_ (mV)	1.47 [0.62–3.57]	1.53 [1.00–1.96]	1.48 [0.86–1.69]
S amplitude in lead V_1_ (mV)	0.74 [0.15–1.93]	1.06 [0.23–1.46]	0.60 [0.44–1.75]
R amplitude in lead V_5_ or V_6_ + S amplitude in lead V_1_ (mV)	2.31 [0.77–4.42]	2.52 [1.29–3.42]	2.08 [1.30–3.44]
P wave amplitude in lead II (mV)	0.10 [0.05–0.15]	0.10 [0.05–0.15]	0.08 [0.05–0.20]
P wave duration in lead II (s)	0.10 [0.06–0.12]	0.06 [0.04–0.10]	0.08 [0.08–0.08]
Morris index (s·mm)	0.01 [0.00–0.08]	0.01 [0.00–0.03]	0.02 [0.00–0.02]
Notched P wave in lead II, *N* (%)	2 (12)	0 (0)	0 (0)

Blood test	(*N* = 7)	(*N* = 4)	(*N* = 1)
BNP (pg/mL)	46.8 [15.8–112.3]	64.9 [19.4–130.7]	79.6 [79.6–79.6]

Echocardiography	(*N* = 10)	(*N* = 7)	(*N* = 2)
Normal left ventricular contraction, *N* (%)	8 (80)	7 (100)	1 (50)
Ejection fraction (%)	68 [46–73]	67 [62–74]	55 [46–63]
Left atrial dimension (mm)	29.3 [23.2–45.6]	32.7 [27.9–37.9]	34.0 [31.1–36.9]
E/A ratio	0.80 [0.43–1.69]	1.11 [0.58–1.70]	1.02 [0.88–1.15]
Deceleration time of E velocity (ms)	196.6 [131.8–225.9]	201.5 [151.7–250.9]	207.3 [180.0–234.6]
E velocity (m/s)	0.51 [0.34–0.80]	0.55 [0.42–1.11]	0.51 [0.36–0.66]
A velocity (m/s)	0.60 [0.32–0.86]	0.66 [0.47–1.00]	0.49 [0.40–0.57]
E/e’ ratio (septal)	9 [8–16]	11 [8–14]	5 [5–5]
e’ velocity (septal) (m/s)	0.06 [0.03–0.08]	0.08 [0.04–0.11]	0.09 [0.07–0.10]

Holter ECG	(*N* = 1)	(*N* = 1)	(*N* = 1)
Number of normal beats/total heartbeats (%)	1.00 [1.00–1.00]	1.00 [1.00–1.00]	1.00 [1.00–1.00]
Number of supraventricular ectopies/total heartbeats (%)	0.00 [ 0.00–0.00]	0.00 [0.00–0.00]	0.00 [0.00–0.00]
Number of ventricular ectopies/total heartbeats (%)	0.00 [0.00–0.00]	0.00 [0.00–0.00]	0.00 [0.00–0.00]
AF burden/recording time (%)	0.00 [0.00–0.00]	0.00 [0.00–0.00]	0.00 [0.00–0.00]

*Note:* Data are presented as median [range] or *N* (%).

We further pursued the factors affecting the variability of AF risk estimation. In 149 patients in Study 2, BNP had been measured in 28 patients, ultrasound echocardiography had been performed in 50 patients, and Holter ECG had been performed in 15 patients. Diseases and treatments which were not eligible for AI‐based AF risk estimation, BNP level, and left ventricular diastolic dysfunction tended to increase in proportion to the magnitude of level change (Table 3). In 30 participants in Study 1, BNP had been measured in 12 patients, echocardiography had been performed in 19 patients, and Holter ECG had been performed in 3 patients. BNP level tended to increase in proportion to the magnitude of level change (Table 4).

### 3.2 Effect of Electrode Placement and Noise Contamination

**FIGURE 5:**
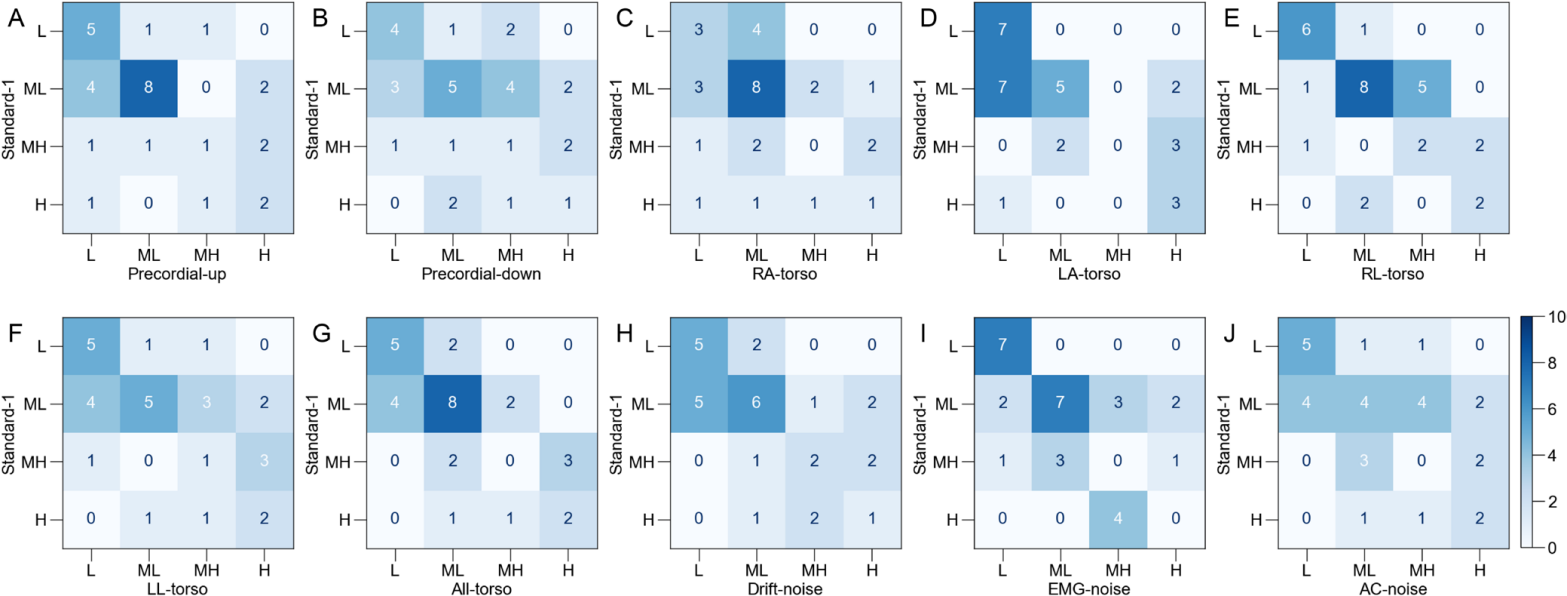
Confusion matrices for the evaluation of the effects of electrode placement and noise contamination. The vertical axes represent Standard‐1, and the horizontal axes represent other conditions. The abbreviations for the conditions are listed in Table 1. The results of the AF risk estimation were expressed as four levels: Low (L), middle‐low (ML), middle‐high (MH), and high (H).

We compared the changes in the estimated AF risk due to changes in the recording conditions in Study 1. The confusion matrices are shown in Figure 5, and the quadratic‐weighted kappa values are listed in Table 5. The changes per participant are shown in Table 6.

**TABLE 5:** Quadratic‐weighted kappa values against the changes in conditions in Study 1.

Conditions		Quadratic weighted kappa value [95% confidence interval]	
No.	Abbreviation		
2	Precordial‐up	0.47	[0.11–0.75]
3	Precordial‐down	0.33	[0.02–0.59]
4	RA‐torso	0.35	[−0.03–0.65]
5	LA‐torso	0.61	[0.29–0.82]
6	RL‐torso	0.63	[0.33–0.83]
7	LL‐torso	0.53	[0.25–0.73]
8	All‐torso	0.69	[0.47–0.83]
9	Drift‐noise	0.59	[0.35–0.76]
10	EMG‐noise	0.55	[0.28–0.73]
11	AC‐noise	0.51	[0.22–0.72]
12	Standard‐2	0.47	[0.07–0.77]

*Note:* The abbreviations for the conditions are listed in Table 1.

**TABLE 6:** Changes in estimated AF risk per participant under various conditions in Study 1.

Condition		Standard‐1	Precordial‐up	Precordial‐down	RA‐torso	LA‐torso	RL‐torso	LL‐torso	All‐torso	Drift‐noise	EMG‐noise	AC‐noise	Standard‐2
Participant	#1	ML	L	L	MH	H	ML	MH	MH	MH	ML	H	ML
	#2	L	L	ML	ML	L	L	L	L	L	L	L	L
	#3	L	MH	MH	L	L	ML	ML	ML	ML	L	MH	H
	#4	L	L	L	L	L	L	L	L	L	L	L	L
	#5	ML	L	L	MH	ML	L	ML	ML	ML	ML	ML	ML
	#6	ML	ML	ML	ML	L	ML	L	L	L	ML	L	L
	#7	ML	H	ML	ML	L	ML	ML	ML	H	MH	MH	ML
	#8	ML	ML	L	ML	L	ML	L	L	ML	L	L	ML
	#9	ML	L	H	H	L	ML	ML	L	ML	ML	L	ML
	#10	ML	ML	MH	ML	L	MH	ML	L	ML	H	H	MH
	#11	H	L	ML	MH	H	H	ML	H	MH	MH	H	H
	#12	MH	MH	L	ML	H	L	H	H	MH	ML	ML	ML
	#13	ML	H	MH	ML	ML	ML	MH	ML	ML	ML	ML	ML
	#14	MH	L	ML	ML	ML	MH	MH	H	H	ML	ML	ML
	#15	MH	ML	MH	L	ML	MH	L	ML	ML	ML	ML	ML
	#16	L	L	L	ML	L	L	MH	ML	ML	L	L	MH
	#17	MH	H	H	H	H	H	H	ML	MH	L	H	MH
	#18	L	ML	MH	ML	L	L	L	L	L	L	ML	L
	#19	H	H	ML	ML	H	ML	H	MH	MH	MH	ML	MH
	#20	MH	H	H	H	H	H	H	H	H	H	H	H
	#21	L	L	L	L	L	L	L	L	L	L	L	L
	#22	ML	ML	ML	ML	L	MH	MH	ML	ML	ML	MH	H
	#23	ML	ML	MH	L	ML	MH	ML	ML	L	MH	ML	MH
	#24	H	MH	MH	L	L	ML	MH	ML	ML	MH	MH	ML
	#25	L	L	L	ML	L	L	L	L	L	L	L	L
	#26	ML	L	H	ML	ML	ML	L	MH	L	L	ML	ML
	#27	ML	ML	ML	ML	H	MH	H	ML	H	H	MH	ML
	#28	ML	ML	MH	L	ML	ML	L	ML	L	MH	MH	ML
	#29	ML	ML	ML	L	L	MH	H	ML	L	ML	L	L
	#30	H	H	H	H	H	H	H	H	H	MH	H	H

*Note:* The abbreviations for the conditions are listed in Table 1. The results of the AF risk estimation were expressed as four levels: low (L), middle‐low (ML), middle‐high (MH), and high (H). The color is darkest at H and becomes lighter towards L.

**FIGURE 6:**
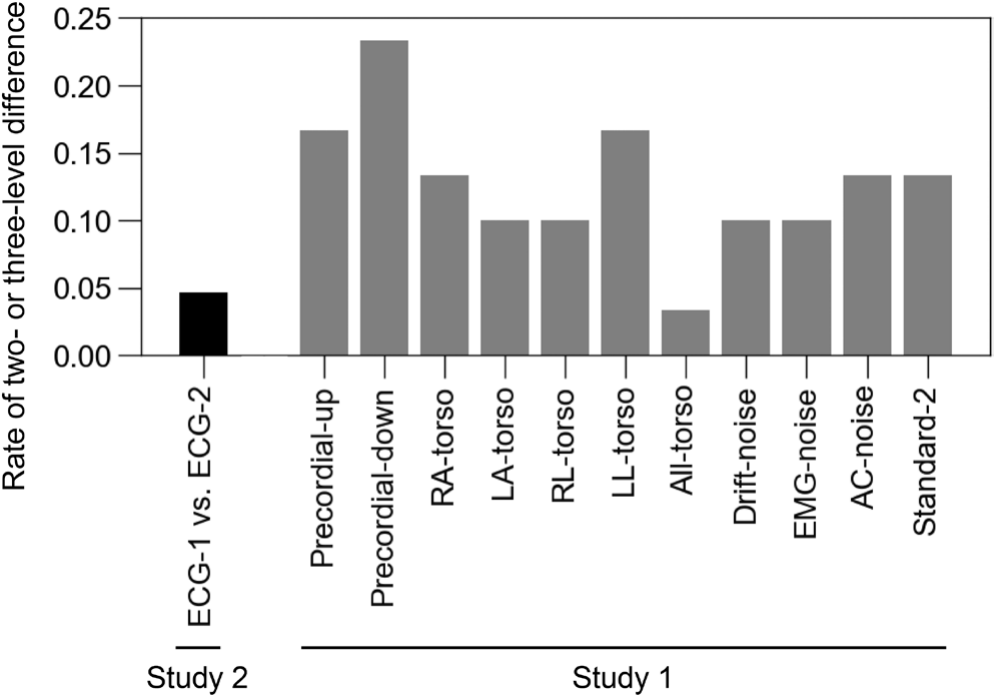
Rate of two‐ or three‐level change in estimated AF risk under various conditions. The differences between ECG‐1 and ECG‐2 in Study 2 and a comparison with Standard‐1 in Study 1 are plotted. The abbreviations for the conditions are listed in Table 1.

Because the reproducibility study identified that a one‐level change might occur in short‐term reproducibility, we evaluated the frequency of two‐ or three‐level changes under each condition (Figure 6). The Precordial‐up, Precordial‐down, and LL‐torso exhibited a higher rate of two‐ or three‐level difference than Standard‐2. In contrast, most of the replacements of limb leads to the torso and the presence of noise contamination did not increase the incidence of two‐ or three‐level differences.

## 4 Discussion

### 4.1 Main Findings

This study evaluated the temporal variability of AI‐based AF risk estimation using a commercially available ECG machine and the variation in results due to the measurement conditions. The results were expressed as four‐class classification and evaluated using the quadratic kappa coefficient. The quadratic kappa values were 0.72 for the reproducibility within 4 min and 0.47 for the reproducibility over 15 min. A deviation of one classification level was considered acceptable, whereas deviations exceeding one tier were considered indicative of nonreproducibility. The rate of participants within one level of error was 95% in the reproducibility test within 4 min and 87% in the reproducibility test over 15 min. With respect to the recording conditions, upward or downward displacement of the precordial electrodes and repositioning of the left leg electrode to the torso were associated with an increased incidence of two‐ or three‐level discrepancies in classification. This effect was not observed with other configurations of limb lead placement on the torso or in the presence of noise contamination.

### 4.2 Temporal Variability of Electrical Conduction in the Heart

Although ECG has been recognized as a reproducible examination [7], it may include slight but significant temporal changes. This study identified that a small temporal change was recognized by the AI‐based classification. The AI model used composite binary images created from the waveforms of the 12 leads [6]. Thus, the waveform may have changed at 4‐ or 15‐min intervals. As the AI model did not explain the basis of its classification, it was difficult to recognize the difference between 1st and 2nd ECG in this study.

To the best of our knowledge, there are no reports on the reproducibility of conventional 12‐lead ECG within 15 min. Several studies have reported the reproducibility or diurnal variation of the minute parts of ECG waveforms. Stafford et al. recorded the signal‐averaged P‐wave twice consecutively and evaluated its reproducibility in patients with PAF and controls. They reported that the P‐wave duration was reproducible but that the frequency domain and spatial velocity analyses were poorly reproducible [13]. Dilaveris et al. reported the mean hourly values of P‐wave duration, P area, and PR interval showed circadian variations in healthy participants using a 12‐lead Holter ECG [14]. Abe et al. also reported that late potentials per half‐hour revealed dynamic daily variations in patients with Brugada syndrome using a Holter‐based signal‐averaged ECG system [15]. These studies revealed that the change in the ECG was detectable in certain indices derived from the averaged waveforms of 100–200 beats or 30–60 min of recording.

It has been widely considered that temporal variability may be one factor of arrhythmogenicity. Thus, the ECG waveform may be more susceptible to change in hearts predisposed to AF, at least in part. It has been reported that the beat‐to‐beat morphological variability of P waves is larger [16] and that cardiac restitution is steeper [17] in patients with AF than in controls. Another study comparing the 1‐h period preceding the onset of paroxysmal AF with the prior 1‐h period revealed that P‐wave duration variability was greater in the period closer to the onset of AF than in the more remote period [18]. In this context, cases exhibiting large temporal variability in AI‐based classifications may involve other arrhythmogenicities that are undetectable by the current AI algorithm. However, this hypothesis warrants further investigation in future studies.

In this study, the quadratic weighted kappa value was lower in comparison with the 15‐min interval than with the 4‐min interval. This finding might indicate that the longer time difference between 2 ECG recordings contributed to the larger variability. We investigated the participants' characteristics in order to find features that might cause a difference in estimated AF risk. We found that the magnitude of level change in estimated AF risk tended to associate with a high BNP level. In addition, the AF risk estimation might be influenced by diseases and treatments which were not eligible for AI‐based AF risk estimation, and left ventricular diastolic dysfunction in reproducibility over 15 min. These points need to be investigated further in the future.

If the ECG waveform had variability, the training data for developing the AI algorithm would have included participants with the various physiological states at the time of recording. The current AI model was developed using a single ECG per participant; thus, it may overweight physiological variability. An AI model that uses multiple ECG recordings from a single participant may help mitigate the impact of within‐participant variability and enable more robust AF risk estimation. Combining multiple AF risk estimation results—for example, adopting an averaged or the worst AF risk level—may improve estimation accuracy. We should find a better usage in the future.

### 4.3 Effect of Electrode Placement and Noise Contamination

In this study, the upward or downward displacement of the precordial electrodes evoked a high rate of two‐ or three‐level difference. It is known that incorrect placement of precordial electrodes affects the morphology of ECG waveforms in the precordial leads [19]. A previous study revealed that misplacement of precordial electrodes can occur even among experienced ECG technicians [10]. Another study asked various medical staff members to indicate the positions of V_1_–V_6_ on a diagram and found that incorrect electrode placement occurred frequently [11]. This study indicates that accurate placement of precordial electrodes is critical for reliable AI‐based AF risk estimation. Particular attention should be paid to quality control for accurate electrode placement to estimate AF risk correctly.

### 4.4 Limitation

This study has several limitations. First, the sample size was small. Second, we did not directly compare AF risk estimation with a thorough assessment of ECG waveform changes or cardiac excitation using other precise techniques. Third, we only evaluated two time points for reproducibility in different participants. Repeated evaluation of temporal changes in the same participant would help to obtain additional information. Fourth, to evaluate very short‐term reproducibility, data were retrospectively collected from hospital laboratory records. As patients underwent multiple ECG examinations, the possibility of patient‐ or measurement‐related bias cannot be excluded, potentially affecting reproducibility. Fifth, we evaluated a limited variety of electrode placements and noise contamination. Nevertheless, our findings underscore the importance of avoiding the incorrect placement of precordial electrodes.

## 5 Conclusions

AI‐based AF risk estimation demonstrates temporal variability. The displacement of the precordial electrodes was found to influence the consistency of AI‐based AF risk estimation.

## Author Contributions

S.H. and T.S. conceived and designed the study. M.A., S.T., and I.K. recruited the patients in Study 1. S.H. recorded ECG signals in Study 1. M.O. extracted the ECG data in Study 2. S.H. and T.S. obtained the background information, analyzed the data, and drafted the manuscript. M.A., M.O., S.T., and I.K. reviewed and edited the manuscript. All the authors approved the final version of the manuscript.

## Funding

The authors have nothing to report.

## Ethics Statement

Approval of the Research Protocol: This study was approved by the Ethics Committee of the Institute of Science, Tokyo (No. I2024‐046).

## Consent

(Study 1) All the participants provided written informed consent. (Study 2) N/A.

## Conflicts of Interest

The authors declare no conflicts of interest.

## Data Availability

The data that support the findings of this study are available on request from the corresponding author. The data are not publicly available due to privacy or ethical restrictions.
